# Suicide Reduction in Schizophrenia via Exercise (SUnRISE): study protocol for a multi-site, single-blind, randomized clinical trial of aerobic exercise for suicide risk reduction in individuals with schizophrenia

**DOI:** 10.1186/s13063-020-04788-z

**Published:** 2020-10-21

**Authors:** Katie Beck-Felts, Marianne Goodman, Luz H. Ospina, Melanie Wall, Joseph McEvoy, Lars F. Jarskog, Jacob S. Ballon, Matthew N. Bartels, Richard Buchsbaum, Richard P. Sloan, T. Scott Stroup, David Kimhy

**Affiliations:** 1 Department of Psychiatry, Icahn School of Medicine at Mount Sinai, One Gustave L. Levy Place, Box 1230, New York, NY 10029 USA; 2 MIRECC, James J. Peters VA Medical Center, Bronx, NY USA; 3 Department of Psychiatry, Columbia University, New York, NY USA; 4 Department of Psychiatry and Health Behavior, Georgia Regents University, Augusta, GA USA; 5 Department of Psychiatry, University of North Carolina, Chapel Hill, NC USA; 6 Department of Psychiatry and Behavioral Sciences, Stanford University, Palo Alto, CA USA; 7 Department of Rehabilitation Medicine, Albert Einstein College of Medicine/Montefiore Medical Center, Bronx, NY USA

**Keywords:** Schizophrenia, Suicide, Exercise, Aerobic fitness, VO_2_max

## Abstract

**Background:**

Suicide risk among individuals with schizophrenia (SZ) is intractably high, with over 40% of individuals attempting to take their own lives during their lifetime and an estimated 5–10% completing suicide. At present, available pharmacological and psychotherapeutic treatments offer limited risk reduction benefits, and thus, there remains an urgent need to explore novel interventions that will ameliorate this risk. Aerobic exercise (AE) has been shown to improve a number of predictors of suicide risk (e.g., depressed mood, sleeping difficulties). As individuals with SZ display a highly sedentary lifestyle, AE may reduce suicide risk.

**Methods:**

Employing a multi-site, single-blind, randomized clinical trial design, we will examine the impact of AE on risk for suicide and related variables in individuals with SZ. Participants will be randomized to one of two 12-week exercise interventions: AE or a stretching and toning (ST) control intervention. Primary outcome measures will include suicide risk (Columbia Suicide Severity Rating Scale, C-SSRS) and aerobic fitness (VO_2_max), along with additional measures of suicide risk, mood, emotion regulation, sleep, cognition, and physical activity, with assessments completed at baseline and after 6 and 12 weeks of interventions.

**Discussion:**

It is hypothesized that AE will reduce suicide risk among individuals with SZ. This study may offer support for a more efficacious treatment method for this population in addition to the pre-existing pharmacological and psychotherapeutic treatment regimens.

**Trial registration:**

Clinicaltrials.gov, NCT03270098. Registered on September 1, 2017.

## Background

People with schizophrenia (SZ) are at an increased risk for suicide, with approximately 40–50% of individuals attempting to take their own lives during their lifetime and an estimated 5–10% completing suicide [1–3]. Furthermore, suicide attempts among individuals with SZ are often more violent and lethal compared to individuals without a diagnosis, suggesting a higher intent to die [4]. This highly elevated risk represents a serious public health concern and an important target for interventions.

Treatment approaches aiming to diminish suicide risk in individuals with SZ have centered primarily on pharmacotherapy and psychotherapy. Available pharmacological treatments have been found to offer only limited benefits to ameliorate these risks [5, 6]. Clozapine has demonstrated some effectiveness in reducing suicidal behavior in patients with SZ [7], and other atypical antipsychotics have been found to be promising, but more evidence is needed [8]. Similarly, evidence for the efficacy of psychotherapy is limited—a small RCT comparing cognitive behavioral therapy for suicide (CBTs; *n* = 25) vs. treatment-as-usual (*n* = 24) among individuals with psychosis found the CBT group improved on primary outcome measures of suicidal ideation, and on secondary outcomes of hopelessness related to suicide probability, depression, psychotic symptoms, and self-esteem [9]. However, systematic analyses focusing on the benefits of psychotherapy on suicide risk in SZ are lacking [10]. Thus, there remains an urgent unmet need to identify novel approaches to reduce suicide risk in people with SZ.

A number of risk factors have been identified as significant predictors of completed suicide among individuals with SZ including depressive mood, sleep difficulties, poor cognition, more severe positive and negative symptoms, and the number of previous hospitalizations [11–15]. Additionally, a recent review and meta-analysis has implicated a number of factors related to increased suicide risk in SZ including hopelessness, physical comorbidity, substance use, poor medication adherence, family history of psychiatric illness, and higher intelligence quotient [15].

Consistent with these findings, sedentary behavior is highly prevalent in people with SZ, with rates far exceeding those in the general population [16]. Studies in SZ have consistently shown higher rates of sedentary behavior, lower physical activity, and cardiovascular fitness, compared to non-clinical populations [17–19]. Using actigraph measurements, our group has found individuals with SZ spend 81% of their wake time sedentary, which was significantly inversely correlated with aerobic fitness (AF), consistent with previous reports [20–22]. Similarly, Strassnig et al. found that 98% of SZ subjects in their study (*n* = 117) displayed low age-adjusted AF [23].

A recent meta-analysis of 14 studies (*n* = 80,856 individuals) indicated a significant inverse association between physical activity and suicidal ideation [24]. Additionally, a number of meta-analyses indicate that SZ patients engage in lower levels of physical activity than healthy controls, which is subsequently significantly associated with a number of predictors of suicide risk including depressed mood and more severe cognitive symptoms [22, 25–27]. However, at present, there are no studies examining directly the impact of AE on suicide risk in individuals with SZ.

Recent reviews and meta-analyses indicate AE training is effective in increasing AF in individuals with SZ, with VO_2_max-indexed improvements ranging from 8 to 38% [17, 25, 28–30]. In our pilot RCT, subjects undergoing a 12-week, 3×/week, 1-h AE training program improved their VO_2_max scores by 3.82 mL/kg/min (SD = 3.21), an 18% improvement, vs. a minimal change in the “treatment-as-usual” control group (M = − 0.48, SD = 3.07; − 0.5%; F = 12.24, *p* = .002; *n* = 26), indicating a large effect size (Cohen’s *d* = 1.06) [31, 32].

Examination of the putative neurobiology underlying suicide, select neurotrophins and inflammation markers show potential association with suicide risk, particularly brain-derived neurotrophic factor (BDNF)—a meta-analysis of 16 studies indicated lower serum BDNF levels in SZ vs. healthy controls, including in drug-naïve patients [33]. Similarly, a review by Buckley et al. found 9 of 11 SZ studies reported reduced serum BDNF vs. controls [34]. These reductions appear to impact multiple brain regions including the dorsolateral prefrontal cortex, anterior cingulate, and the inferior and superior temporal gyri [35]. Studies assessing individuals who have previously attempted suicide indicate both lower levels of serum and plasma BDNF compared to non-attempters [36–39]. However, a few studies have demonstrated no difference in serum BDNF levels between suicide attempters vs. non-attempters, nor correlations between plasma BDNF and clinical symptoms [40–43]. To date, the only study that examined BDNF and suicidality in SZ found no difference in serum BDNF levels between suicide attempters and non-attempters [44]. However, available studies have a number of limitations including the use of clinical indicators of suicide rather than research measures and extended latency between evaluations of suicide risk and biomarkers. Additionally, few studies examined the longitudinal links between BDNF and suicide risk in individuals with SZ using parallel assessments. Given previous literature has suggested BDNF as a potential biomarker for suicide risk, further research is necessary to elucidate the role of BDNF in suicide risk in SZ.

In summary, there remains an urgent unmet need to identify novel approaches to target suicide risk in people with SZ. Converging lines of animal, basic human, and clinical research support, the scientific premise for this project, suggesting AE, a novel intervention, may potentially ameliorate suicide risk in people with SZ. This study takes advantage of the infrastructure of a large, ongoing NIMH R01-funded project investigating the effects of AE on cognition in individuals with SZ. The relatively large size of the study cohort (*n* = 160) would permit examination of the impact of multiple covariates and biomarkers, as well as intervention characteristics. Such knowledge will inform development of more granular and precise models of the mechanisms underlying suicide risk in this population.

## Study AIMS

The aim of this study is to examine the impact of AE training on suicide risk and related predictors in individuals with SZ.

*Hypothesis 1.1*: At 12 weeks, subjects in the AE training will significantly reduce their suicide risk compared to controls.

*Hypothesis 1.2*: Increases in aerobic fitness will predict reduction in suicide risk.

*Hypothesis 2.1*: At 12 weeks, subjects in the AE training will significantly improve their depressed mood, sleep quality and quantity, and executive functioning compared to controls.

*Hypothesis 2.2*: AE-induced improvements in suicide risk from baseline to 6 weeks to 12 weeks will be mediated by decreases in depressed mood and improvement in sleep quality and quantity, and executive functioning.

## Methods/design

**Fig. 1:**
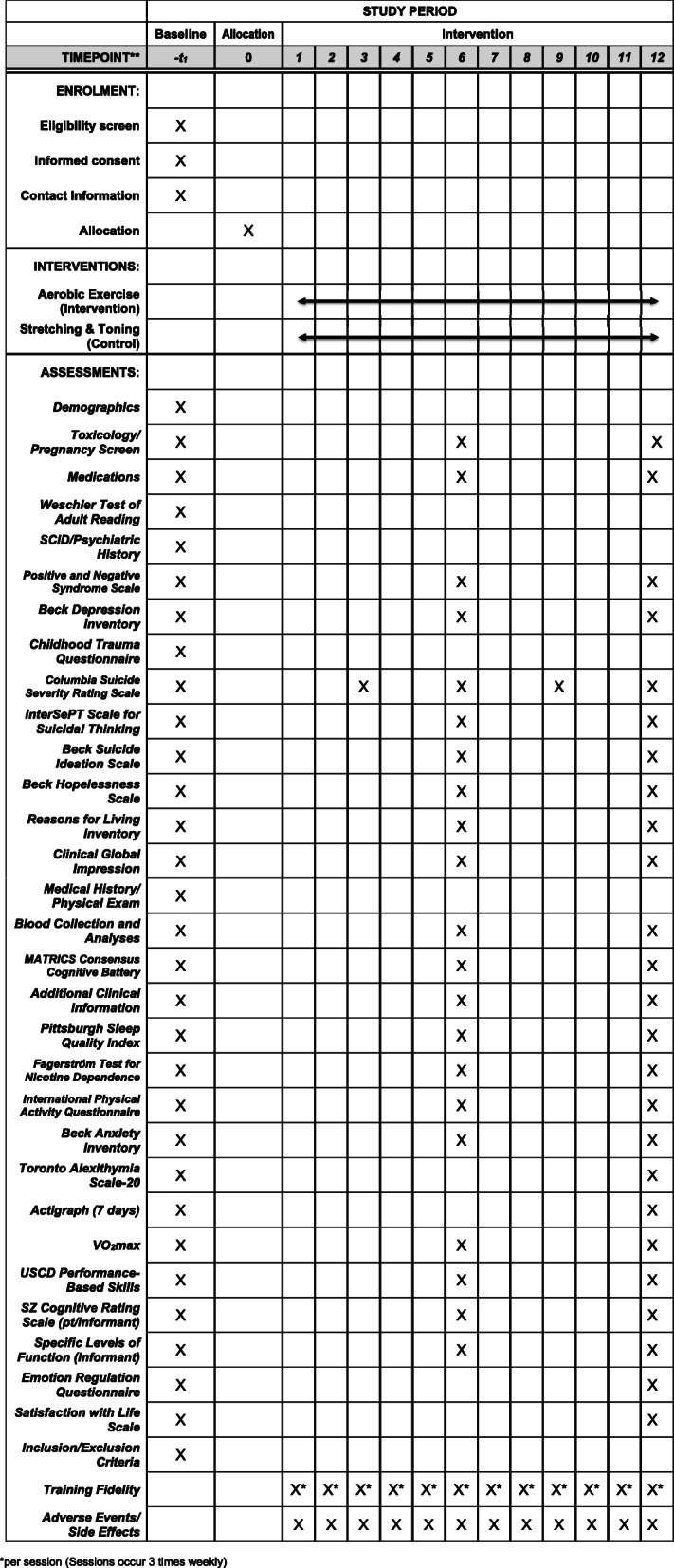
Detailed study timeline

This is a multisite, single-blind, randomized clinical trial testing an intervention to improve suicide risk in individuals with psychosis. The Consolidated Standards of Reporting Trials (CONSORT) guidelines will be followed to ensure the standardized conduct and reporting of research. This protocol has been prepared in accordance with the Standard Protocol Items: Recommendations for Interventional Trials (SPIRIT) guidelines. The checklist is presented in Additional file 1. Details regarding the study procedure are outlined in Fig. 1. Amendments to the current protocol will be reported to the IRBs, trial participants, and The Trial Journal.

The interventions and data collection will take place at four geographically diverse sites in the USA: Icahn School of Medicine at Mount Sinai (New York), University of North Carolina at Chapel Hill (North Carolina), Augusta University (Georgia), and Stanford University (California). Each site has access to an exercise facility and spaces dedicated for blood drawing, biological samples analyses and banking, and VO_2_max assessments.

Recruitment of 160 eligible participants will be conducted independently across the four sites. Each site PI will contact clinicians in their healthcare systems and local communities and inform them about the study so that they may inform their eligible patients of the study. Brochures will be left at various relevant outpatient facilities. Interested individuals may speak with a member of the research team who will describe the study in more detail and will invite them to enroll. A short screening interview will be conducted by a researcher remotely by phone to ensure that participants satisfy inclusion criteria.

The number of screens, eligible subjects, and reasons for non-participation will be tracked using the CONSORT guidelines. In an earlier pilot study, a similar procedure was employed with 77% of approached individuals recruited. Refusals were related primarily to logistical/physical health reasons. Subjects’ retention was high, with only 1/16 AE subjects discontinuing due to AE program dislike. In the ongoing parent R01-funded protocol, to date, 71% of approached individuals consented to participate in the study. Formal recruitment for the parent study began in May of 2018. Formal recruitment for this amended study began in November of 2019.

### Eligibility criteria

Inclusion Criteria: 18–55 years of age; DSM-V diagnoses of SZ, schizoaffective disorder, or schizophreniform disorder; taking an antipsychotic medication for at least 8 weeks and on current dose for 4 weeks and/or taking an injectable depot antipsychotic with no change in last 3 months; medically cleared by a physician to take part in VO_2_max tests and AE/ST training; capacity to understand all the potential risks and benefits of the study; and consented to participate in the parent R01 study (“Improving Cognition via Exercise in Schizophrenia”; MH110623; PIs: Kimhy & Stroup) which aims to assess the impact of AE on cognitive and daily functioning in people with SZ.

Exclusion Criteria: DSM-IV diagnosis of alcohol/substance abuse (except nicotine) within the last month or a diagnosis of alcohol/substance dependence (except nicotine) within the last 6 months; initiation of anti-depressants, mood stabilizers, or other medications known to impact cognition in previous 4 weeks or any changes in doses during this period; history of seizures/head trauma with loss of consciousness (> 10 min) resulting in cognitive sequelae; significant clinical abnormalities in physical examination, lab assessments, or ECG; neurological/medical conditions that could interfere with study participation (e.g., AIDS, unstable cardiac disease, stuttering); BMI ≥ 40; untreated hyper- or hypothyroidism; pregnant/nursing; serious homicidal risk (past 6 months); “moderate” or more severe conceptual disorganization (PANSS ≥ 4); poor English reading ability (WTAR< 7); and participation in a study with cognitive assessment in the past 3 months.

This study has been approved by the institutional review board of the Icahn School of Medicine at Mount Sinai, New York, NY; University of North Carolina, Chapel Hill, NC; Stanford University, Stanford, CA; and Augusta University, Augusta, GA. As part of the informed consent process, the rationale of the study and its methods will be described in detail and all questions will be answered. All participants will be asked to provide written informed consent before participation.

## Baseline measures

Baseline assessments follow an initial phone screen and occur over the course of 3 visits. Primary outcome measures of the current study are assessed via administration of The Columbia Suicide Severity Rating Scale (C-SSRS) by a blinded, trained rater. Four additional measures of suicidality supplement the C-SSRS: InterSePT Scale for Suicidal Thinking, Beck Suicide Ideation Scale, Beck Hopelessness Scale, and Reasons for Living Inventory.

In accordance with the parent study protocol, secondary measures will also be collected at this time. Descriptive data will include gender, age, ethnic and racial background, employment status, education level, current living arrangements, current medications, menstrual cycle information (if applicable), and contact information for the participant’s primary mental health care provider. Several questionnaires will be administered at this time including the Beck Depression Inventory (BDI), Beck Anxiety Inventory (BAI), Childhood Trauma Questionnaire (CTQ), Pittsburgh Sleep Quality Index (PSQI), Fagerström Test for Nicotine Dependence (FTND), International Physical Activity Questionnaire (IPAQ), Toronto Alexithymia Scale (TAS-20), Emotion Regulation Questionnaire (ERQ), and The Satisfaction with Life Scale (SLS). The Structured Clinical Interview for DSM-IV-TR Axis I Disorders (SCID-I) will be administered by a trained clinician to confirm diagnoses of psychiatric illnesses. Severity of psychotic symptoms will be assessed using the Positive and Negative Syndrome Scale (PANSS). A medical physician associated with the study at each site will collect a medical history and conduct a medical examination. A urine Toxicology Screen and a pregnancy test (if applicable) will be taken along with blood samples. Aerobic fitness as indexed by VO_2_max will be assessed with the use of electro-magnetically braked cycle ergometers by a cardiologist associated with each site.

## Randomization

After signing informed consent and undergoing baseline assessment of primary and secondary outcome measures, eligible participants will be assigned 1:1 randomly via computer to one of two 12-week, 3×/week, 1-h interventions: (1) aerobic exercise intervention (AE) or (2) a stretching and toning control intervention (ST). Participants will receive intervention assignment via a secure telephone call.

## Blinding

While participants, exercise trainers, and coordinators cannot be blinded, the assessors of primary and secondary outcomes are blind to participants’ allocations by the following procedures. Suicide risk as well as assessments of aerobic fitness, cognitive, and daily functioning will be conducted by blinded evaluators (cognitive raters and medical professionals). The raters and medical professionals are not involved in the allocation process or the exercise intervention. Before the outcome measurements begin, participants, coordinators, and exercise trainers are instructed not to reveal any information regarding the allocation or exercise intervention. Data from the pilot study for the parent R01 study indicates that the risk for un-blinding is minimal—only 2 of 41 subjects were un-blinded with no status disclosures during any of the cognitive tests, clinical and functioning interviews. In the ongoing parent R01-funded study, only one subject was unblinded to date, with no status disclosures during any of the cognitive tests, clinical and functioning interviews.

## Interventions

Patients assigned to AE or ST receive treatment as usual from their treatment providers. The interventions are conducted at local site-affiliated exercise facilities, held in small groups between 1 and 4 subjects, and led by a certified exercise trainer. A non-blinded member of the research team will be present during all exercise sessions to set-up and manage equipment (e.g., heart rate monitors), as well as record behavioral data. All subjects are encouraged to hydrate and take breaks as needed.

Both the AE and ST treatment groups will exercise for 1 h three times a week. This frequency was chosen based on previous studies of AE in SZ and informed by federal and ACSM guidelines which recommend 150 min of moderate-intensity AE per week over 3 days [45, 46]. Such activities expend 3–5.9 times the energy expended at rest and are defined as activities in which the subject is able to talk while engaging in the activity. A group exercise format with a trainer was chosen over individual independent training based on findings indicating lower attrition rates in AE RCTs using trainers [25].

At the beginning of each AE session, the trainer will lead participants through a 10-min warm-up period. The following AE portion of the session will consist of participants using AE equipment. Each site is equipped with identical equipment including a LifeSpan TR 1200i treadmill, LifeSpan S1 stationary bike, NordicTrack E7.5 Elliptical machine, and an Xbox video-game console with a Kinect motion-sensing device that will run Your Shape Fitness Evolved 2012 (Ubisoft Inc., San Francisco, CA), an interactive whole-body fitness activity software. The Xbox Kinect was selected due to the American Heart Association recommendation of the console and similar equipment as tools for promoting physical activity. Previous studies demonstrated the device’s capability of stimulating moderately intense aerobic activity in adults, with significantly higher enjoyment ratings as compared to “traditional” exercises [47–49]. Participants will be free to choose the equipment they use, with encouragement from the trainer to change activities throughout the session to diversify physical exercise routines. Each session will end with a trainer-led 5-min cool-down period.

Intensity of training in the AE condition will be individualized to each participant based on his/her maximal heart rate as determined during the VO_2_max test conducted at baseline. During week 1, training will aim to maintain the participant’s HR at 60% of their max HR. This goal will increase to 65% in week 2, 70% in week 3, and 75% for the remainder of the 12 weeks. HR will be monitored using Polar V800 HR monitors (a digital watch connected to a Bluetooth-enabled H10 sensor strap) that participants will wear during sessions [50]. The monitors will be programmed to emit a soft beeping sound when the participant’s HR drops below the individually-targeted weekly AE intensity level. In this case, the trainer will encourage him/her to achieve their target goal. At session’s end, the data is uploaded from the monitors to a computer and analyzed using Polar Flow Sync software.

The stretching and toning (ST) condition is designed to replicate the variables of the AE condition such as schedule, duration, facilities, format, trainer contact, and social interactions without affecting aerobic fitness. Similarly to the AE training sessions, ST sessions will begin with a 10-min warm-up period led by the trainer. The following 45-min of each session will consist of a mixture of static stretching positions, low-impact toning exercises, and yoga- and tai chi-inspired routines using the Xbox Kinect. ST exercises are recommended by American College of Sports Medicine (ASCM) guidelines to improve flexibility [45, 46]. To maintain interest, new exercises will be introduced every week. While static stretching may result in limited transient HR increases, it provides limited aerobic fitness benefits [51–54]. Just as in the AE condition, each ST session will end with a trainer-led 5-min cool-down period.

Intensity of training in the ST condition will also be individualized per participant based on his/her maximal heart rate as determined during the VO_2_max test conducted at baseline. However, the training will aim to keep the participant’s HR below 60% of their max HR. HR will be monitored using Polar V800 HR monitors (a digital watch connected to a Bluetooth-enabled H10 sensor strap) that participants will wear during sessions [50]. The monitors will be programmed to emit a soft beeping sound when the participant’s HR exceeds the individually calculated ST maximum intensity level. In this case, the trainer will encourage him/her to rest until his/her HR lowers adequately. At session’s end, the data is uploaded from the monitors to a computer and analyzed using Polar Flow Sync software.

### Training fidelity

In both training conditions, training fidelity will be monitored and indexed by (1) the number of sessions attended and (2) the number of minutes per session the participant trained with his/her HR above (for AE condition) or below (for ST condition) the designated training intensity. The in-session training intensity is included to address the possibility that participants may attend the sessions but exercise too lightly (for AE condition) or too intensely (for ST condition).

### Training safety

At the end of each week of training, potential minor and moderate exercise-related adverse events will be recorded (in addition to serious adverse events; SAE). These may include musculoskeletal/soft tissue (muscle soreness; muscle pull/strain; joint pain, falls), cardiovascular (fatigue; dyspnea; angina or chest pains that are resolved with rest), and skin-related side-effects (blisters; chafing). In cases chest pain ensues, the medical physician and principal investigator associated with each site will be called to ensure the safety of the participant. Previous reports indicate the risk of adverse events as part of AE-related intervention studies is small [55]. The screening procedure, the use of a “start low and go slow” strategy, the close monitoring of exercise intensity, and the presence of a certified exercise trainer will minimize the risk of SAE and adverse events.

## Outcome measures

**Fig. 2:**
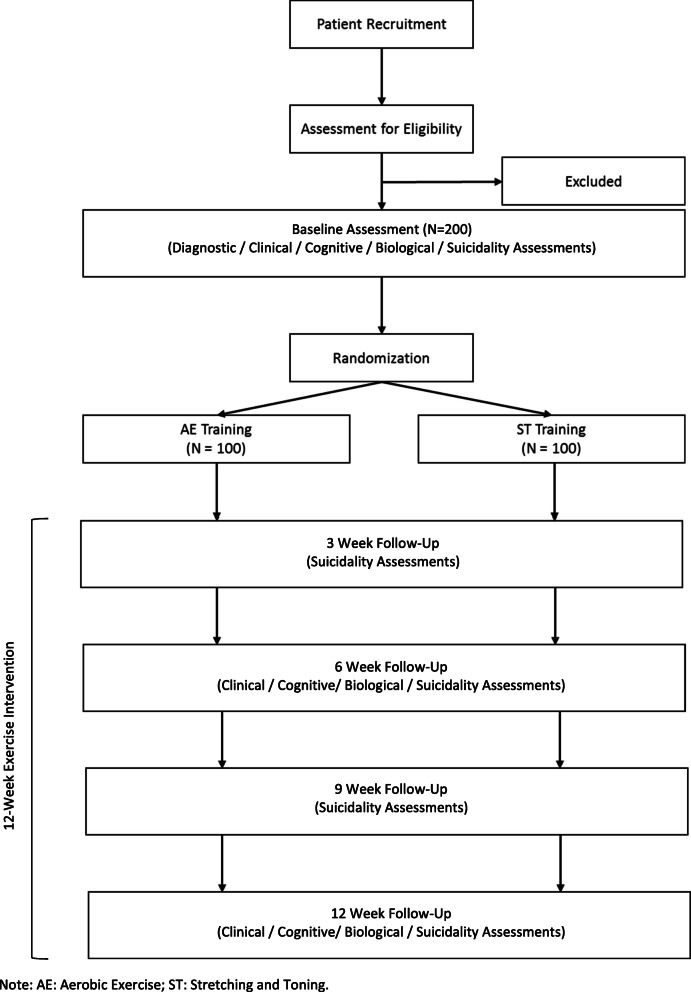
Study protocol

All outcome measures will be identically assessed by the same examiners throughout a subject’s participation in the study. See Fig. 2 for a full assessment schedule. The following procedures will be administered by trained clinical raters, research coordinators, and medical professionals adhering to standardized protocols.

### Suicide risk

Blinded Clinical Raters will administer suicide risk measures to all participants during baseline, 6-week, and 12-week research visits. As part of the protocol, the clinical raters will obtain certification in the administration of the suicide, clinical, and other measures prior to initiating the study, as well as annually. For assessments of suicide risk, all current and future raters will complete the certificate training for the C-SSRS. Additionally, Dr. Goodman, an expert on suicide risk, will be available for inquiries/supervision regarding assessment of suicide risk throughout the study duration.

The Columbia Suicide Severity Rating Scale (C-SSRS) will serve as a primary outcome measure of suicide risk [56]. The C-SSRS is a 12-item, semi-structured interview that measures 4 suicide risk-related domains: ideation severity, ideation intensity, behavior, and lethality. To address potential safety concerns associated with recruitment of individuals with higher suicide risk, all participants will also be administered the C-SSRS by phone at 3 and 9 weeks after baseline. Additional secondary measures of suicide risk will include the InterSePT Scale for Suicidal Thinking (ISST), the Beck Suicide Ideation Scale (BSIS), Beck Hopelessness Scale (BHS), and the Reasons for Living Inventory (RLF) [57–60]. All the suicide risk measures will be administered at baseline, and after 6 and 12 weeks in parallel to the assessments of the parent R01-funded protocol.

### Secondary measures

Data on demographics, medical history, and diagnoses will be obtained at baseline. Diagnoses will be established using the Structured Clinical Interview for DSM-IV Axis I Disorders (SCID-I) by a well-trained clinical rater. A medical doctor will conduct a full medical examination of each patient and take a thorough medical history. Demographic information and additional clinical information will be collected by a member of the research team.

Data on medications, sleep, menstrual phase, and smoking will be collected at baseline, and after 6 and 12 weeks by a member of the research team. Antipsychotic medications will be indexed by chlorpromazine equivalence. As other medications may impact outcomes, medication use/changes will be tracked using three broad classes: (1) antidepressants, (2) mood stabilizers, and (3) other medications. As indices allowing direct comparisons of medications are not available, for each class, the use of relevant medications at baseline will be documented using a dummy variable (1, 0), along with increases, no change, decreases, or discontinuation at follow-ups (1, 0, − 1, or − 2, respectively). PRN use of medications that may impact cognition (e.g., antihistamines for allergies) during 48 h prior to cognitive and AF tests will be assessed, and the tests will be postponed by 24 h. A similar strategy will be used to handle medications known to impact biomarkers (e.g., SSRIs on BDNF). Sleep during the 24 h prior to cognitive and AF tests will be assessed using an abbreviated version of The Pittsburgh Sleep Quality Index (PSQI), a measure used extensively in SZ studies [61–63]. If a subject reports substantial acute sleep loss that is likely to influence outcomes (< 4 h sleep), the evaluations will be postponed until sleep is restored [64]. Smoking frequency and dependence as well as the number of cigarettes smoked in the 4 h prior to tests (nicotine half-life = ~ 2 h) will be recorded using the Fagerström Test for Nicotine Dependence (FTND) [65–67].

### Psychiatric symptoms

Symptom severity will be assessed with the Positive and Negative Syndrome Scale (PANSS) along with the Beck Depression Inventory (BDI) and Beck Anxiety Inventory (BAI). These assessments will be administered by blinded clinical raters at baseline, 6 weeks, and 12 weeks.

### Cognitive functioning

The composite score of the MATRICS Consensus Cognitive Battery (MCCB) will stand as the primary cognitive functioning assessment and will be administered by blinded clinical raters at baseline, 6 weeks, and 12 weeks. The WTAR will additionally be utilized at baseline to assess reading ability for eligibility.

### Daily functioning

The total score of the Specific Levels of Functioning scale (SLOF; informant version) will serve as the chief daily functioning outcome measure, along with The UCSD Performance-based Skills Assessment (UPSA) and the Schizophrenia Cognition Rating Scale (SCoRS). These measures of daily functioning will be administered by blinded clinical raters at baseline, 6 weeks, and 12 weeks.

### Aerobic fitness

Aerobic fitness will be determined according to participants’ VO_2_max. The VO_2_max tests are completed by an exercise physiologist under the supervision of a physician using electro-magnetically braked cycle ergometers at baseline, 6 weeks, and 12 weeks. A blinded cardiopulmonary physiology expert will review each test to assess for the quality of the test and to ensure that no medical issues arise that would prevent the participant to safely continue exercise.

### Physical activity

Data gathered by an accelerometer (wGT3x-BT) worn on the wrist of the non-dominant hand will provide a measure of objective physical activity [68]. This measure will be performed at the baseline and 12-week follow-up visits. The participant will wear the device for seven consecutive days. Data will be sampled at a frequency of 80 Hz for this duration. Established cut points will be used to determine physical activity [69]. Self-reported physical activity will be assessed with the International Physical Activity Questionnaire [70].

### Biospecimens

A urine sample will be collected by a member of the research team at baseline, 6 weeks, and 12 weeks to be used for toxicology screen (Alere iCup) [71]. Blood samples will be collected by a certified phlebotomist. All samples will be collected between 9 and 10 am to minimize potential diurnal-related variability. The samples will be analyzed for fasting hemoglobin A1c and a lipid panel as well as several biomarkers of cognitive change including neurotrophins and inflammation. Assessment of neurotrophins and inflammation markers will include serum BDNF (R&D Systems, Minneapolis, MN), Nerve Growth Factor - NGF Emax Immuno Assay Systems (Promega, Sunnyvale, CA), Neurotrophin-3 - NT-3 Emax Immuno Assay Systems (Promega, Sunnyvale, CA), Neurotrophin-4 - NT-4 DuoSet ELISA Development Kit (R&D System, Minneapolis, MN), Tumor Necrosis Factor alpha (TNF-α) - MILLIPLEX MAP Human High Sensitivity Immunology Multiplex Assay (Millpore, Billerica, MA), Interleukin-6 (IL6) - MILLIPLEX MAP Human High Sensitivity Immunology Multiplex Assay (Millpore, Billerica, MA), and C-Reactive Protein (CRP) - Cobas Integra 400 Plus (Roche Diagnostics, Indianapolis, IN).

### Emotion regulation, trauma, and quality of life

Questionnaires will be administered by a member of the research team at baseline and 12-week follow-up to collect information regarding emotion regulation (ERQ), alexithymia (TAS-20), childhood trauma (CTQ; only administered at baseline), and the participant’s satisfaction with their life (SWL).

## Safety measurements/adverse events

### Exercise safety measures

At the end of each week of training, potential exercise-related minor and moderate adverse events will be recorded and monitored in addition to serious adverse events (SAE). These may include musculoskeletal/soft tissue (muscle soreness; muscle pull/strain; joint pain, falls), cardiovascular (fatigue; dyspnea; angina or chest pains that are resolved with rest), and skin-related side-effects (blisters; chafing). If chest pain ensues, the medical physician and principal investigator associated with each site will be called to ensure the safety of the participant. Previous reports indicate the risk of adverse events as part of AE-related intervention studies is small [55]. The screening procedure, the use of a “start low and go slow” strategy, the close monitoring of exercise intensity, and the presence of a certified exercise trainer will minimize the risk of SAE and adverse events.

### Suicide risk safety

To address potential safety concerns associated with recruitment of individuals with higher suicide risk, all participants will also be administered a suicide risk assessment (i.e., C-SSRS) by phone at 3 and 9 weeks after baseline, in addition to the suicide risk assessments scheduled during research visits at baseline, 6 weeks, and 12 weeks. All assessments will capture suicidal symptoms from the last administration of the instrument. The raters will consult with the site PI regarding the detection of any suicidality, including passive suicidality. Any subject with a C-SSRS score equal or greater than “4” (Active Suicidal Ideation with Some Intent to Act, without Specific Plan) will require a consultation with Dr. Scott Stroup, the project’s Medical Director, and Dr. Marianne Goodman, the project’s suicide expert. Any subject that scores a “5” (Active Suicidal Ideation with Specific Plan and Intent) and judged to be actively suicidal will require urgent clinical evaluation for the possibility of hospitalization, and he/she will be escorted to the closest ER by study staff. Dr. Goodman has extensive clinical experience treating veterans at high risk for suicide and is available by cell phone 24/7, should clinical consultation regarding study participants be necessary.

### Data monitoring

A NIMH-approved Data and Safety Monitoring Board (DSMB) was established in December of 2017 to oversee the parent study. The members of the DSMB have no financial, scientific, or other conflict of interest with the study and have no affiliations with any of the participating institutions. All materials, discussions, and proceedings of the DSMB are completely confidential and objective. The DSMB will meet every 6 months, or as deemed necessary. Meetings shall be closed to the public because discussions may address confidential patient data. Meetings are attended by the principal investigators and members of his staff. All serious adverse events (SAE) will be reported to the DSMB in addition to the IRB and NIMH.

### Auditing

The current study is monitored by the NIMH Office of Clinical Research (OCR)‘s Clinical Research Education, Support, and Training (CREST) program. (https://www.nimh.nih.gov/funding/clinical-research/nimh-clinical-research-education-support-and-training-crest-program.shtml). The CREST Program aims to establish accurate and verifiable clinical research study data, as well as compliance with Good Clinical Practice and human subjects safety regulations. As part of the program, NIMH staff conduct annual site visits. Additionally, the study is monitored by a Data and Safety Monitoring Board (DSMB) that meets twice annually during the duration of the study.

### Sample size calculations

A power analysis was considered for determining sample size. However, a power analysis was ultimately determined not to be appropriate for the current trial due to the lack of direct data on the relationship between aerobic exercise and reduction in suicide risk. Additionally, recruitment and sample size for this protocol is associated with the ongoing parent R01-funded protocol.

The sample size for the parent R01-funded study is 200 outpatient individuals with SZ. This sample size was determined based on a focus on cognition as the primary outcome, specifically the MCCB total composite score. Prior literature suggests average scores of individuals with SZ fall between 1 and 2.5 standard deviations lower than MCCB averages. Therefore, a 0.50 standard deviation was chosen as a meaningful target effect size for powering the trial. The pilot for the parent study found a large effect size for the AE intervention group compared to controls suggesting a 0.893 standard deviation improvement in the intervention group compared to controls. The parent study sample size calculations assumed a loss-to-follow-up rate of 20% with 80 participants remaining in each arm (100 × 0.8). According to these calculations, the parent study is powered to detect moderate or larger effect size difference with > 80% power. The current study retains confidence that the sample size will provide adequate power to detect a clinically meaningful effect size for suicide risk as the primary outcome.

### Data analyses

All primary analyses will be carried out on an intent-to-treat (ITT) basis, i.e., all subjects randomized will be analyzed according to the treatment they were assigned regardless of adherence to treatment. Level of fidelity to training intensity, accounting for adherence, will be controlled for in these analyses. All statistical tests will be two-sided with critical value α = 5%.

Mixed effects regression will be used to model suicide risk as a function of time (0, 6, 12 weeks), treatment group (AE or ST), interaction of time by treatment group, and site (1, 2, 3, or 4). The efficacy of AE to improve suicide risk in people with SZ will be represented by the statistical significance of the time by treatment group interaction. The effect of AE on mood, sleep, and executive functions will be tested by conducting similar analyses, i.e., mixed effects regression across 0, 6, and 12 weeks.

Mediators in the relationship between AE and suicide risk will be identified by multi-step process. A mediation effect will be estimated by taking the expected value of the total effect of AE on changes in suicide risk and subtracting the effect when changes in mood, sleep, and executive functioning have been controlled. These mediation effects will be tested by fitting a Structural Equation Model (SEM) with change in depressed mood, sleep, and executive function from baseline to 6 weeks as mediators of the relationship between AE treatment and change in suicide risk from baseline to 6 weeks as well as later changes in suicide risk from 6 to 12 weeks using MPlus version 8. The mediator-outcome relationship can be confounded by other time-varying covariates (e.g., BMI changes), and these will also be adjusted for in the model.

Change in suicide risk from baseline will be modeled using mixed effect regression predicted by contemporaneous change in aerobic fitness from baseline as measured by VO_2_max tests (at 6 and 12 weeks), controlling for treatment group and other covariates. The ability of aerobic fitness improvement to predict reduction in suicide risk will be represented by a statistically significant positive regression coefficient for change in aerobic fitness.

Demographic and other biologically relevant baseline variables (e.g., sex, depression, and smoking status) will be included as control variables if there is evidence of randomization imbalance in them. Multiple imputation will be used to handle missing data using the MI and MIANALYZE SAS procedures.

### Confidentiality and data management

All study documents will be deidentified and labeled using participant identification codes. The identification code key, contact information, consent forms, and medical documents with personal health information will be kept confidential. Hard copies are kept in secure, locked cabinets. Electronic copies will be stored in password-protected computers. Only IRB authorized individuals will be able to access study data.

## Discussion

Individuals with SZ are at an elevated risk for committing and completing suicide [1–3]. Current pharmacological and psychotherapeutic treatment approaches provide limited benefits to ameliorate suicide risk in this population. Aerobic exercise may help mitigate many of the predictors of suicide risk. Therefore, engaging individuals with SZ in AE may be a beneficial treatment approach.

### Trial status

This trial (protocol version 1.7, January 4, 2020) is currently active. Recruitment of participants for the parent study started in May of 2018. Recruitment for the Suicide Reduction in Schizophrenia via Exercise amendment started in November of 2019. Recruitment of participants is scheduled to be completed July 31, 2021.

## Supplementary information

**Additional file 1.** SPIRIT 2013 Checklist: Recommended items to address in a clinical trial protocol and related documents*.

## Data Availability

Anonymized data from the study will be available from the author after the trial has ended.

## Abbreviations

ACSM: American College of Sports Medicine
AE: Aerobic exercise
AF: Aerobic fitness
BAI: Beck Anxiety Index
BDI: Beck Depression Index
BDNF: Brain-derived neurotrophic factor
BHS: Beck Hopelessness Scale
BSS: Beck Suicide Ideation Scale
BMI: Body mass index
CBTs: Cognitive behavioral therapy for suicide
C-SSRS: Columbia Suicide Severity Rating Scale
CTQ: Childhood Trauma Questionnaire
DSM: Diagnostic and Statistical Manual of Mental Disorders
DSMB: Data Safety and Monitoring Board
ERQ: Emotion Regulation Questionnaire
FTND: Fagerström Test for Nicotine Dependence
HR: Heart rate
ICE: Improving Cognition via Exercise
IPAQ: International Physical Activity Questionnaire
IRB: Institutional Review Board
ISST: The InterSePT Scale for Suicidal Thinking
ITT: Intent-to-treat
MCCB: MATRICS Consensus Cognitive Battery
NIMH: National Institute of Mental Health
PANSS: Positive and Negative Symptom Scale
PRN: Pro re nata (“as needed”)
PSQI: Pittsburgh Sleep Quality Index
RCT: Randomized clinical trial
RFL: Reasons for Living Inventory
SAE: Serious adverse event
SCID: Structured Clinical Interview for DSM-V Axis 1 Disorders
SCORS: Schizophrenia Cognition Rating Scale
SEM: Structural Equation Model
SLOF: Specific Levels of Functioning scale
SMI: Serious mental illness
SSRI: Serotonin reuptake inhibitors
ST: Stretching and toning
SWL: Satisfaction with Life Questionnaire
SZ: Schizophrenia
TAS-20: Toronto Alexithymia Scale
UPSA: UCSD Performance-based Skills Assessment

## References

[1] Roy A (1986). Depression, attempted suicide, and suicide in patients with chronic schizophrenia. Psychiatr Clin North Am.

[2] Miles CP (1977). Conditions predisposing to suicide: a review. J Nerv Ment Dis.

[3] Palmer BA, Pankratz VS, Bostwick JM (2005). The lifetime risk of suicide in schizophrenia: a reexamination. Arch Gen Psychiatry.

[4] Harkavy-Friedman JM, Restifo K, Malaspina D, Kaufmann CA, Amador XF, Yale SA (1999). Suicidal behavior in schizophrenia: characteristics of individuals who had and had not attempted suicide. Am J Psychiatry.

[5] Siris SG (2001). Suicide and schizophrenia. J Psychopharmacol.

[6] Khan A, Khan SR, Leventhal RM, Brown WA (2001). Symptom reduction and suicide risk among patients treated with placebo in antipsychotic clinical trials: an analysis of the food and drug administration database. Am J Psychiatry.

[7] Mehlum L, Dieserud G, Ekeberg O, Groholt B, Mellesdal L, Walby F (2006). NIPH systematic reviews: executive summaries. In: Prevention of suicide Part 1: Psychotherapy, drug treatment and electroconvulsive treatment.

[8] Wasserman D, Rihmer Z, Rujescu D, Sarchiapone M, Sokolowski M, Titelman D (2012). The European Psychiatric Association (EPA) guidance on suicide treatment and prevention. Eur Psychiatry.

[9] Tarrier N, Kelly J, Maqsood S, Snelson N, Maxwell J, Law H (2014). The cognitive behavioural prevention of suicide in psychosis: a clinical trial. Schizophr Res.

[10] Pompili M, Girardi P, Ruberto A, Tatarelli R (2004). Toward a new prevention of suicide in schizophrenia. World J Biol Psychiatry.

[11] Bornheimer LA (2019). Suicidal ideation in first-episode psychosis (FEP): examination of symptoms of depression and psychosis among individuals in an early phase of treatment. Suicide Life Threat Behav.

[12] Clapham E, Boden R, Brandt L, Jonsson EG, Bahmanyar S, Ekbom A, et al. Suicide ideation and behavior as risk factors for subsequent suicide in schizophrenia: a nested case-control study. Suicide Life Threat Behav. 2018; Available from: https://www.ncbi.nlm.nih.gov/pubmed/30073690. 10.1111/sltb.12499 30073690

[13] Li SX, Lam SP, Zhang J, Yu MWM, Chan JWY, Chan CSY, et al. Sleep disturbances and suicide risk in an 8-year longitudinal study of schizophrenia-Spectrum disorders. Sleep. 2016;39(6):1275–82. 10.5665/sleep.5852. 10.5665/sleep.5852 PMC4863217 27091530

[14] Canal-Rivero M, Lopez-Morinigo JD, Setien-Suero E, Ruiz-Veguilla M, Ayuso-Mateos JL, Ayesa-Arriola R (2018). Predicting suicidal behaviour after first episode of non-affective psychosis: the role of neurocognitive functioning. Eur Psychiatry.

[15] Cassidy RM, Yang F, Kapczinski F, Passos IC (2018). Risk factors for suicidality in patients with schizophrenia: a systematic review, meta-analysis, and meta-regression of 96 studies. Schizophr Bull.

[16] Vancampfort D, Guelinckx H, Probst M, Ward PB, Rosenbaum S, Stubbs B (2015). Aerobic capacity is associated with global functioning in people with schizophrenia. J Ment Heal.

[17] Rosenbaum S, Watkins A, Teasdale S, Curtis J, Samaras K, Kalucy M (2015). Aerobic exercise capacity: an important correlate of psychosocial function in first episode psychosis. Acta Psychiatr Scand.

[18] Vancampfort D, Probst M, Knapen J, Carraro A, De Hert M (2012). Associations between sedentary behaviour and metabolic parameters in patients with schizophrenia. Psychiatry Res.

[19] Nyboe L, Vestergaard CH, Moeller MK, Lund H, Videbech P (2015). Metabolic syndrome and aerobic fitness in patients with first-episode schizophrenia, including a 1-year follow-up. Schizophr Res.

[20] Kimhy D, Vakhrusheva J, Bartels MN, Armstrong HF, Ballon JS, Khan S (2014). Aerobic fitness and body mass index in individuals with schizophrenia: implications for neurocognition and daily functioning. Psychiatry Res.

[21] Faulkner G, Cohn T, Remington G (2006). Validation of a physical activity assessment tool for individuals with schizophrenia. Schizophr Res.

[22] Stubbs B, Firth J, Berry A, Schuch FB, Rosenbaum S, Gaughran F (2016). How much physical activity do people with schizophrenia engage in? A systematic review, comparative meta-analysis and meta-regression. Schizophr Res.

[23] Strassnig M, Brar JS, Ganguli R (2011). Low cardiorespiratory fitness and physical functional capacity in obese patients with schizophrenia. Schizophr Res.

[24] Vancampfort D, Hallgren M, Firth J, Rosenbaum S, Schuch FB, Mugisha J, et al. Physical activity and suicidal ideation: a systematic review and meta-analysis. J Affect Disord. 2018;225:438–48. 10.1016/j.jad.2017.08.070. 10.1016/j.jad.2017.08.070 28858658

[25] Firth J, Cotter J, Elliott R, French P, Yung AR. A systematic review and meta-analysis of exercise interventions in schizophrenia patients. Psychol Med. 2015;45(7):1343–61. 10.1017/S0033291714003110. 10.1017/S0033291714003110 25650668

[26] Dauwan M, Begemann MJ, Heringa SM, Sommer IE (2016). Exercise improves clinical symptoms, quality of life, global functioning, and depression in schizophrenia: a systematic review and meta-analysis. Schizophr Bull.

[27] Vancampfort D, Probst M, Scheewe T, De Herdt A, Sweers K, Knapen J (2013). Relationships between physical fitness, physical activity, smoking and metabolic and mental health parameters in people with schizophrenia. Psychiatry Res.

[28] Gorczynski P, Faulkner G. Exercise therapy for schizophrenia. Cochrane Database Syst Rev. 2010;(5):CD004412 Available from: https://www.ncbi.nlm.nih.gov/pubmed/20464730. 10.1002/14651858.CD004412.pub2 PMC4164954 20464730

[29] Scheewe TW, Backx FJ, Takken T, Jorg F, van Strater AC, Kroes AG (2013). Exercise therapy improves mental and physical health in schizophrenia: a randomised controlled trial. Acta Psychiatr Scand.

[30] Scheewe TW, van Haren NE, Sarkisyan G, Schnack HG, Brouwer RM, de Glint M (2013). Exercise therapy, cardiorespiratory fitness and their effect on brain volumes: a randomised controlled trial in patients with schizophrenia and healthy controls. Eur Neuropsychopharmacol.

[31] Kimhy D, Vakhrusheva J, Bartels MN, Armstrong HF, Ballon JS, Khan S (2015). The impact of aerobic exercise on brain-derived neurotrophic factor and neurocognition in individuals with schizophrenia: a single-blind, randomized clinical trial. Schizophr Bull.

[32] Armstrong HF, Bartels MN, Paslavski O, Cain D, Shoval HA, Ballon JS, et al. The impact of aerobic exercise training on cardiopulmonary functioning in individuals with schizophrenia. Schizophrenia Res. 2016;173(1-2):116–7. 10.1016/j.schres.2016.03.009. 10.1016/j.schres.2016.03.009 26976498

[33] Green MJ, Matheson SL, Shepherd A, Weickert CS, Carr VJ (2011). Brain-derived neurotrophic factor levels in schizophrenia: a systematic review with meta-analysis. Mol Psychiatry.

[34] Buckley PF, Pillai A, Howell KR (2011). Brain-derived neurotrophic factor: findings in schizophrenia. Curr Opin Psychiatry.

[35] Ray MT, Shannon Weickert C, Webster MJ (2014). Decreased BDNF and TrkB mRNA expression in multiple cortical areas of patients with schizophrenia and mood disorders. Transl Psychiatry.

[36] Priya PK, Rajappa M, Kattimani S, Mohanraj PS, Revathy G (2016). Association of neurotrophins, inflammation and stress with suicide risk in young adults. Clin Chim Acta.

[37] Ambrus L, Lindqvist D, Traskman-Bendz L, Westrin A (2016). Hypothalamic-pituitary-adrenal axis hyperactivity is associated with decreased brain-derived neurotrophic factor in female suicide attempters. Nord J Psychiatry.

[38] Kudinova AY, Deak T, Deak MM, Gibb BE (2019). Circulating levels of brain-derived neurotrophic factor and history of suicide attempts in women. Suicide Life Threat Behav.

[39] Janelidze S, Ventorp F, Erhardt S, Hansson O, Minthon L, Flax J (2013). Altered chemokine levels in the cerebrospinal fluid and plasma of suicide attempters. Psychoneuroendocrinology.

[40] Eisen RB, Perera S, Banfield L, Anglin R, Minuzzi L, Samaan Z. Association between BDNF levels and suicidal behaviour: a systematic review and meta-analysis. Syst Rev. 2015;4:187. 10.1186/s13643-015-0179-z. 10.1186/s13643-015-0179-z PMC4697315 26718989

[41] Cantarelli MD, Nardin P, Buffon A, Eidt MC, Godoy LA, Fernandes BS (2015). Serum triglycerides, but not cholesterol or leptin, are decreased in suicide attempters with mood disorders. J Affect Disord.

[42] Eisen RB, Perera S, Bawor M, Dennis BB, El-Sheikh W, DeJesus J, et al. Exploring the association between serum BDNF and attempted suicide. Sci Rep. 2016;6:25229. 10.1038/srep25229. 10.1038/srep25229 PMC4848497 27121496

[43] Ambrus L, Sunnqvist C, Ekman R, Traskman-Bendz L, Westrin A (2016). Plasma brain-derived neurotrophic factor and psychopathology in attempted suicide. Neuropsychobiology..

[44] Huang TL, Lee CT (2006). Associations between serum brain-derived neurotrophic factor levels and clinical phenotypes in schizophrenia patients. J Psychiatr Res.

[45] Garber CE, Blissmer B, Deschenes MR, Franklin BA, Lamonte MJ, Lee IM, et al. Quantity and quality of exercise for developing and maintaining cardiorespiratory, musculoskeletal, and neuromotor fitness in apparently healthy adults: guidance for prescribing exercise. Med Sci Sports Exerc. 2011;43(7):1334-59. 10.1249/MSS.0b013e318213fefb. 10.1249/MSS.0b013e318213fefb 21694556

[46] 2008 Physical Activity Guidelines for Americans [Internet]. Health. 2008. Available from: www.health.gov/paguidelines.

[47] Lieberman DA, Chamberlin B, Medina E, Franklin BA, Sanner BMH, Vafiadis DK. The power of play: innovations in getting active summit 2011: a science panel proceedings report from the American Heart Association. Circulation. 2011;123(21):2507–16. 10.1161/CIR.0b013e318219661d. 10.1161/CIR.0b013e318219661d 21518980

[48] Lanningham-Foster L, Foster RC, McCrady SK, Jensen TB, Mitre N, Levine JA (2009). Activity-promoting video games and increased energy expenditure. J Pediatr.

[49] Graves LEF, Ridgers ND, Williams K, Stratton G, Atkinson G, Cable NT. The physiological cost and enjoyment of Wii fit in adolescents, young adults, and older adults. J Phys Act Health. 2010;7(3):393–401. 10.1123/jpah.7.3.393. 10.1123/jpah.7.3.393 20551497

[50] Polar V800 Watch with H7 Sensor Strap [Internet]. Lake Success, NY: Polar Inc.; Available from: www.polar.com.

[51] Gladwell VF, Coote JH. Heart rate at the onset of muscle contraction and during passive muscle stretch in humans: a role for mechanoreceptors. J Physiol. 2002;540(Pt 3):1095–102. 10.1113/jphysiol.2001.013486. 10.1113/jphysiol.2001.013486 PMC2290287 11986394

[52] Mojock CD, Kim JS, Eccles DW, Panton LB. The effects of static stretching on running economy and endurance performance in female distance runners during treadmill running. J Strength Cond Res. 2011;25(8):2170–6. 10.1519/JSC.0b013e3181e859db. 10.1519/JSC.0b013e3181e859db 21610517

[53] Nelson AG, Kokkonen J (2013). Elevated metabolic rate during passive stretching is not a sufficient aerobic warm-up. J Sport Heal Sci.

[54] Baker LD, Frank LL, Foster-Schubert K, Green PS, Wilkinson CW, McTiernan A, et al. Effects of aerobic exercise on mild cognitive impairment: a controlled trial. Arch Neurol. 2010;69(1):71–9. 10.1001/archneurol.2009.307. 10.1001/archneurol.2009.307 PMC3056436 20065132

[55] Ory M, Resnick B, Jordan PJ, Coday M, Riebe D, Garber CE, et al. Screening, safety, and adverse events in physical activity interventions: collaborative experiences from the behavior change consortium. Ann Behav Med. 2005;29 Suppl:20–8. 10.1207/s15324796abm2902s_5. 10.1207/s15324796abm2902s_5 15921486

[56] Posner K, Brown GK, Stanley B, Brent DA, Yershova KV, Oquendo MA (2011). The Columbia-Suicide Severity Rating Scale: initial validity and internal consistency findings from three multisite studies with adolescents and adults. Am J Psychiatry.

[57] Lindenmayer JP, Czobor P, Alphs L, Nathan AM, Anand R, Islam Z (2003). The InterSePT scale for suicidal thinking reliability and validity. Schizophr Res.

[58] Beck AT, Steer RA (1991). Manual for the Beck scale for suicide ideation.

[59] Beck AT, Weissman A, Lester D, Trexler L (1974). The measurement of pessimism: the hopelessness scale. J Consult Clin Psychol.

[60] Linehan MM, Goodstein JL, Nielsen SL, Chiles JA (1983). Reasons for staying alive when you are thinking of killing yourself: the Reasons for Living Inventory. J Consult Clin Psychol.

[61] Afonso P, Figueira ML, Paiva T (2014). Sleep-wake patterns in schizophrenia patients compared to healthy controls. World J Biol Psychiatry.

[62] Buysse DJ, Reynolds CF, Monk TH, Berman SR, Kupfer DJ (1989). The Pittsburgh Sleep Quality Index: a new instrument for psychiatric practice and research. Psychiatry Res.

[63] Waters F, Naik N, Rock D (2013). Sleep, fatigue, and functional health in psychotic patients. Schizophr Res Treat.

[64] Belenky G, Wesensten NJ, Thorne DR, Thomas ML, Sing HC, Redmond DP (2003). Patterns of performance degradation and restoration during sleep restriction and subsequent recovery: a sleep dose-response study. J Sleep Res.

[65] Allen MH, Debanne M, Lazignac C, Adam E, Dickinson LM, Damsa C (2011). Effect of nicotine replacement therapy on agitation in smokers with schizophrenia: a double-blind, randomized, placebo-controlled study. Am J Psychiatry.

[66] Benowitz NL, Kuyt F, Jacob P (1982). Circadian blood nicotine concentrations during cigarette smoking. Clin Pharmacol Ther.

[67] Benowitz NL, Jacob P, Jones RT, Rosenberg J (1982). Interindividual variability in the metabolism and cardiovascular effects of nicotine in man. J Pharmacol Exp Ther.

[68] ActiGraphTM wGT3X-BT + ActiLife [Internet]. ActiGraph Corp; 2016. Available from: https://www.actigraphcorp.com/actigraph-wgt3x-bt/.

[69] Trost SG, Loprinzi PD, Moore R, Pfeiffer KA. Comparison of accelerometer cut points for predicting activity intensity in youth. Med Sci Sports Exerc. 2011;43(7):1360–8. 10.1249/MSS.0b013e318206476e. 10.1249/MSS.0b013e318206476e 21131873

[70] Craig CL, Marshall AL, Sjostrom M, Bauman AE, Booth ML, Ainsworth BE (2003). International physical activity questionnaire: 12-country reliability and validity. Med Sci Sport Exerc.

[71] Abbott. Alere iCup/iCup A.D. [Internet]. Abbott Park, Illinois; 2020. Available from: https://www.alere.com/en/home/product-details/icup-a-d-drug-screen.html.

